# Supplementary material for: Dual-comb optomechanical spectroscopy
Source: Nat Commun. 2023 Aug 18;14:5037. doi: 10.1038/s41467-023-40771-3 (PMC10439198; doi:10.1038/s41467-023-40771-3)
Supplement: Supplementary file 1 — Supplementary Information [file 41467_2023_40771_MOESM1_ESM.pdf]

Supplementary Information for

# Dual-comb optomechanical spectroscopy

Xinyi Ren[1]†, Jin Pan[1]†, Ming Yan[1,2,3]*, Jiteng Sheng[1,4]*, Cheng Yang[1], Qiankun Zhang[1], Hui Ma[1], Zhaoyang Wen[1], Kun Huang[1], Haibin Wu[1,4,5,6]* and Heping Zeng[1,2,3,7]*

[1]State Key Laboratory of Precision Spectroscopy, East China Normal University, Shanghai 200062, China

[2]Chongqing Key Laboratory of Precision Optics, Chongqing Institute of East China Normal University, Chongqing 401120, China

[3]Chongqing Institute for Brain and Intelligence, Guangyang Bay Laboratory, Chongqing 400064, China.

[4]Collaborative Innovation Center of Extreme Optics, Shanxi University, Taiyuan 030006, China

[5]Shanghai Research Center for Quantum Sciences, Shanghai 201315, China

[6]Shanghai Branch, Hefei National Laboratory, Shanghai 201315, China

[7]Jinan Institute of Quantum Technology, Jinan, Shandong 250101, China

†These authors contribute equally.

* Corresponding author: myan@lps.ecnu.edu.cn, jtsheng@lps.ecnu.edu.cn, hbwu@phy.ecnu.edu.cn, hpzeng@phy.ecnu.edu.cn

**This PDF file includes:**

Tables 1

Notes 1 to 7

Figures 1 to 9

References 1 to 13

## Supplementary Tables

## Table 1. Comparison of ultrasensitive PAS systems

| Ref. | Method | Sensing type | Gas | λ<br>μm | Detection bandwidth<br>Hz | Spectral elements in a single measurement | $P_o$<br>mW | *t*<br>s | Detection limit<br>ppb | NNEA<br>$cm^{-1}\cdot W\cdot Hz^{-1/2}$ |
|---|---|---|---|---|---|---|---|---|---|---|
| 1 | CW+ wavelength tuning | Quartz tuning fork | $NH_3$ | 1.53 | 0.833 | Single element | 3000 | 1 | 14 | $8.15\times10^{-9}$ |
| 2 | CW+ wavelength tuning | Silicon cantilever optical microphones | $CH_4$ | 1.65 | A few tens | Single element | 14.7 | 1 | 111.2 | $1.2\times10^{-9}$ |
| 3 | CW+ wavelength tuning | Fabry-Perot fiber optic microphones | $CH_4$ | 1.65 | 0.25 | Single element | 14.7 | 1 | 36.45 | $4.4\times10^{-10}$ |
| 4 | CW+ wavelength tuning | Cavity-enhanced PAS | $C_2H_2$ | 1.53 | 62.6 | Single element | 17 | 1<br>1000 | 8.17<br>0.6 | $1.84\times10^{-8}$ |
| 5 | CW+ wavelength tuning | Intra-cavity QEPAS | $N_2O$<br>CO | 4.59 | 0.2 | Single element | 19.5<br>25 | 10 | 0.79<br>0.27 | $1.7\times10^{-10}$<br>$3.8\times10^{-10}$ |
| 6 | CW+ wavelength tuning | Cavity-enhanced QEPAS | $C_2H_2$ | 1.53 | / | Single element | 4 | 300 | 0.3 | $1.1\times10^{-11}$ |
| 7 | CW+ wavelength tuning | Cavity-enhanced QEPAS | $C_2H_2$ | 1.53 | 1 | Single element | 300 | 300 | 0.0005 | $1.7\times10^{-12}$ |
| 8 | CW+ wavelength tuning | Cavity-enhanced CEPAS | $C_2H_2$ | 1.53 | / | Single element | 7.5 | 10 | 0.075 | $1.75\times10^{-12}$ |
| This Work | Dual-comb multiplexing | Optomechanically enhanced | $C_2H_2$ | 1.53 | $0.6\times10^5$ at -3dB<br>$3.4\times10^5$ at -10dB | 40 elements | 150 | 100 | 1.1 | $1.7\times10^{-11}$ |

*t*: measurement time; $P_o$: excitation power.

## Supplementary Notes

### Supplementary Note 1. Generation of EO combs

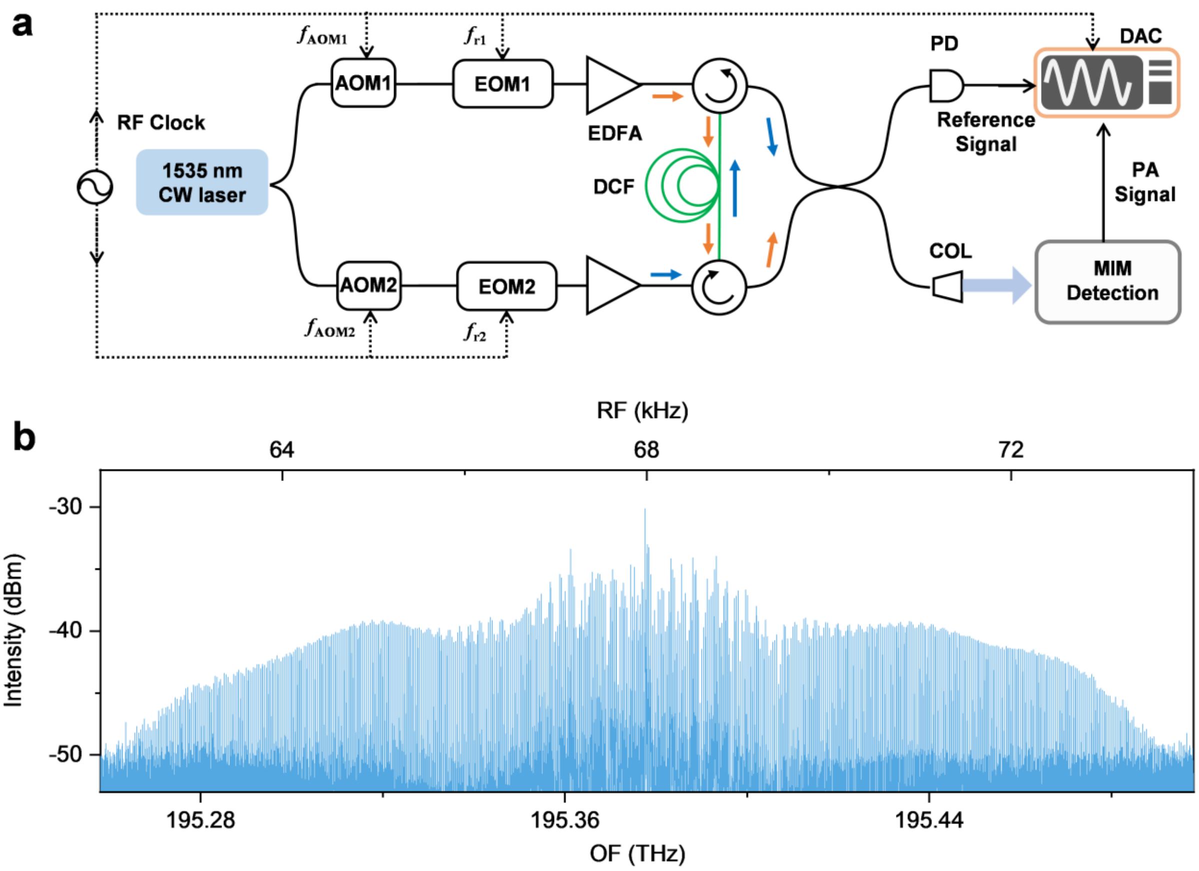


**Fig. 1 | Generation of EO combs. a** Detailed experimental setup for the dual-comb source. **b** Comb-line-resolved dual-comb spectrum measured with a photodetector. AOM, acousto-optic modulator; EOM, electro-optic modulator; DCF, dispersion-compensating fiber; COL, collimator; CIR, circulator; DAC, data acquisition card; EDFA, erbium-doped fiber amplifier; PD, photodetector; MIM, the membrane-in-the-middle sensor.

The two electro-optic (EO) combs are shown in Fig. 1a. The driving frequencies of the two AOMs are set at 100 MHz and 99.932 MHz, respectively, generating an offset frequency of 68 kHz, corresponding to the resonance peak of the MIM sensor. For each comb, a single EOM is used to generate 30-ps light pulses with the repetition rate tunable from 9 kHz to 1 GHz. The initial bandwidth of each comb is about 30 GHz. For spectral broadening, we first boost the comb power

to about 150 mW using an EDFA (for each) and then guide the combs into a 0.2-km-long DCF (YOFC; www.yofc.com). As a result, two flat-top near-infrared combs, spanning 240 GHz (or 2 nm), are generated by wave-breaking in the DCF. In Fig. 1b, a dual-comb spectrum, measured with a photodetector, is displayed in both radio-frequency (RF) and optical frequency (OF) domains. The line spacings of the two combs are 400 MHz and 400 MHz + 20 Hz, respectively. The spectrum in Fig. 1b, containing about 600 comb lines, is used for normalization of the photoacoustic (PA) signal. Note that the dual-comb power could be further boosted with additional EDFAs to improve the minimum detectable concentration. However, one should consider the photothermal noise and the intensity noise induced by the high-power amplifier.

**Supplementary Note 2. The MIM cavity optomechanical system**

In our experiment, the MIM cavity optomechanical system (Fig. 2) is utilized as an ultrasensitive displacement detector. To this end, we inject a 1064-nm frequency-adjustable laser through an EOM along the axis of the MIM cavity. The reflected light signal is extracted through quarter-wave plate (λ/4) and a polarization beam splitter (PBS), and detected by a photodetector (PD). We employ the Pound-Drever-Hall (PDH) method to keep the 1064-nm probing laser resonating with the MIM system[9]. The error signal of the PDH unit is analyzed to reflect the membrane's motion.

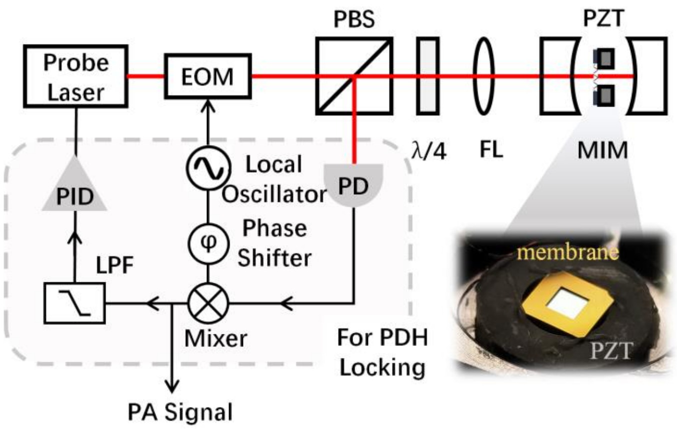


**Fig. 2 | Experimental setup for the MIM detection.** λ/4, quarter-wave plate; FL, focusing lens; PZT, piezoelectric transducer; PID, Proportion integration differentiation controller; LPF, low-pass filter; PA signal, photoacoustic signal. Inset: a nanomechanical membrane attached to the PZT.

In order to realize high sensitivity detection, it is necessary to keep the optomechanical coupling strength maximum. We adjust the position of the membrane in the cavity by changing the voltage of the PZT (Fig. 2 Inset) to optimize the coupling strength. Besides, In the PDH unit (dashed box in Fig. 2), the input laser of the probing field is phase modulated via an EOM driven by a local RF oscillator at a frequency $\Omega_0$ (~3MHz). The modulation frequency $\Omega_0$ is much larger than the cavity amplitude decay rate $\kappa$ ($\sim 2\pi \times 210$ kHz), so that the PDH sidebands are directly reflected from the cavity.

Since our system does not need a long interaction length between the light and molecules, the size of the MIM system (Fig. 3) can be small.

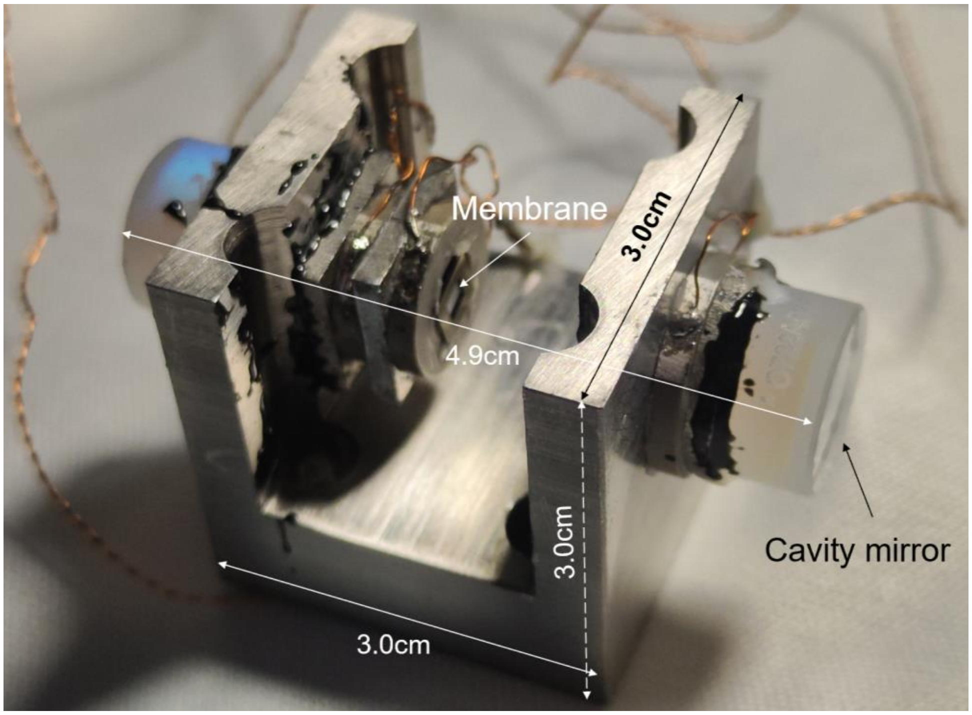


**Fig. 3 | Picture of the MIM system.** The size is 3×3×4.9 $cm^3$.

## Supplementary Note 3. Rapid spectral measurements

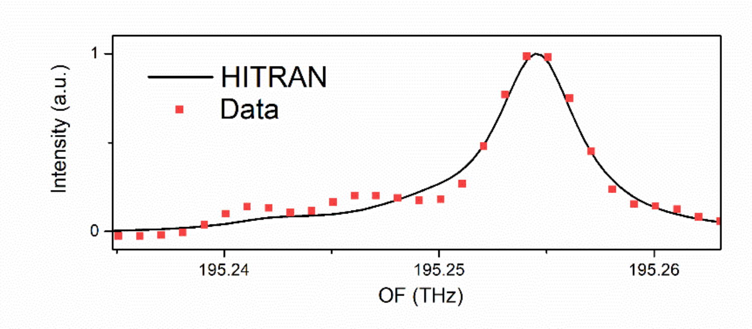


**Fig. 4 | Normalized spectral results of a single measurement ($\Delta f_r$=2 kHz)**.

Fig. 4 exemplifies the spectral results (red squares) acquired in a single measurement for 1% $C_2H_2/N_2$ ($10^5$ Pa, 295 K). The measurement takes 500 μs with a spectral resolution of 1 GHz (or ~8 pm) and a data refresh rate of 2 kHz (i.e., $f_r$~1GHz, $\Delta f_r$=2 kHz). The HITRAN simulation (black curve) is calculated using the spectral parameters of the $^{12}C_2H_2$ $v_1+v_3$ band P(17) transition.

## Supplementary Note 4. High-resolution measurement

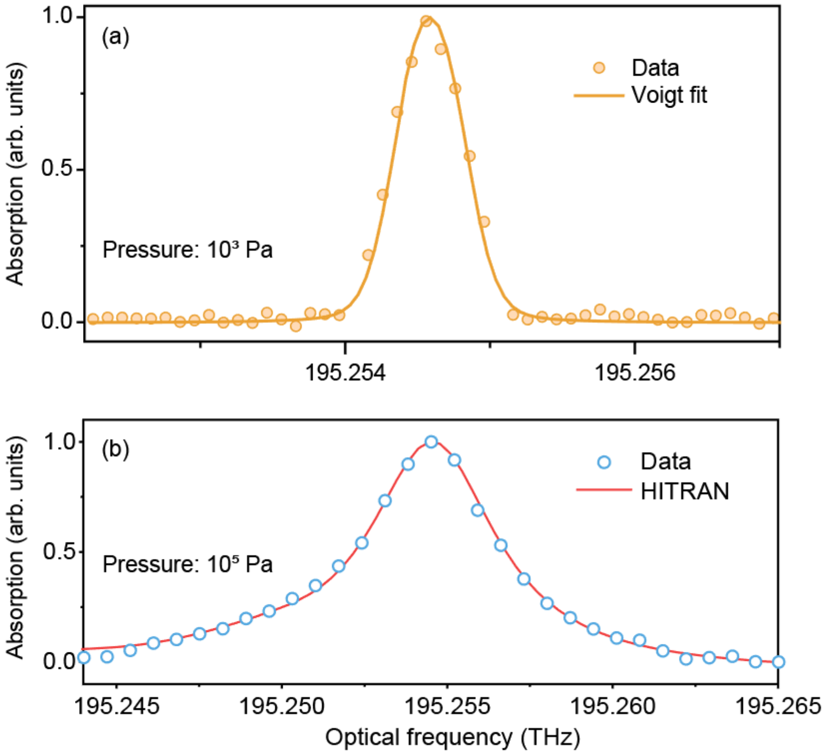


**Fig. 5 | Spectral measurements for the ($v_1$+$v_3$) band P(17) line of $^{12}C_2H_2$ (a) at the pressure of $10^3$ Pa and (b) under atmospheric pressure.**

We measure high-resolution spectra for the ($v_1$+$v_3$) band P(17) line of 50% $^{12}C_2H_2$. The sample pressure is $10^3$ Pa and the temperature is 295 K. The results measured within 2 s are shown in Fig. 5(a). The data points represent the peaks of photoacoustic dual-comb lines (spaced at 100 MHz). We fit the data with a Voigt profile by fixing the Gaussian linewidth at 470 MHz (determined by the Doppler broadening), which gives a Lorentz linewidth of 35 MHz (due to collision broadening). The results measured under atmospheric pressure (Fig. 5(b)) are displayed for comparison.

**Supplementary Note 5. Experimental calibration of the displacement sensitivity**

In the ideal model, the coupling coefficient $g_{\mathrm{m}} = g_{\mathrm{m}}^{\max}$ and the overlap coefficient $\eta_{xy}$ equals to 1. Practically, the two coefficients deviate from the ideal values. In addition, the system sensitivity is also limited by the electronic noise introduced in the PDH locking process, the collision of the membrane and gas molecules under atmospheric pressure, as well as other factors. Therefore, we need to investigate the system sensitivity experimentally.

In order to calibrate the displacement sensitivity of the MIM system, we compare it with a Michelson interferometer which is fed by a laser with a wavelength of 795nm[13]. The experiment setup for this demonstration is shown in Fig. 6. We divide the light into two arms: the probe arm (containing the membrane and the PZT1) and the reference arm (with the high-reflective mirror (HR) fixed at PZT2). The optical powers of the two arms are adjusted to be identical for balanced detection. The detection unit includes two identical photodetectors, PD1 and PD2. The light of the two arms is divided into two parts by a 50:50 beam splitter (BS). Then, we use the signal captured by the balanced detector to lock the interferometer. The optical path of the reference arm is controlled using a PID and piezo servo system, so that the interferometer can sense a tiny change of the membrane displacement (on the order of tens of femtometers).

To experimentally verify the shot-noise-limited displacement sensitivity of our system, we apply a sinusoidal voltage to the PZT1 to ensure a periodic motion of the membrane. The PZT1 attaches to the membrane tightly and the displacement of the membrane's motion should be large enough to be quantified by the Michelson interferometer. We observe the same motion signal in the MIM system and evaluate the signal-to noise ratio (SNR). The noise-floor equivalent displacement can be calculated according to the SNR of the MIM system.

During the measurement, we use a RF signal generator to output the sinusoidal signal at 68 kHz for driving the PZT1 (shown in Fig. 6). As a result, the peak value of the membrane displacement power spectrum is 269.21 fm/$\sqrt{Hz}$ detected by the Michelson interferometer. Under the same condition, the measured SNR for a displacement power spectrum of the MIM system is 47dB so that the noise-floor equivalent displacement is 1.20 fm/$\sqrt{Hz}$. This is an order of magnitude larger than the theoretical value (0.12 fm/$\sqrt{Hz}$). We notice that the electronic noise of nearly 17dB introduced by the mixer and the electrical amplifier used in the PDH locking circuit limit the sensitivity of our system. After excluding the electronic noise, the displacement sensitivity may be optimized to 0.17 fm/√Hz, which is very close to the theoretical calculation.

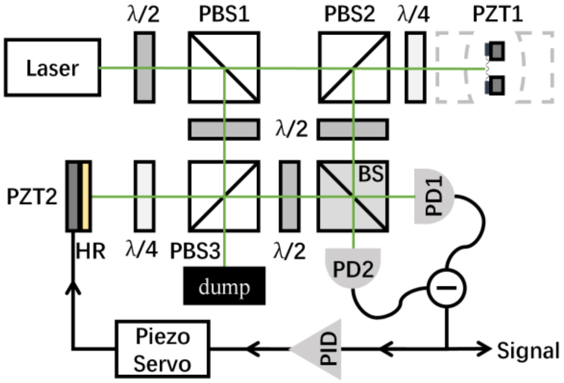


**Fig. 6 | Experimental setup for the Michelson interferometer.** λ/2, half wave plate; λ/4, quarter-wave plate; PBS1, polarization beam splitter used to separates a reference arm; PBS2, polarization beam splitter used to extract the reflected field of the membrane; PZT1, ring piezoelectric transducer used to drive the membrane; PZT2, piezoelectric transducer for scanning the optical path and locking the interferometer; HR, high-reflective mirror; PBS3, polarization beam splitter used to extract the reflected field from the HR; PD1 and PD2, photodetectors on the balanced detector for locking the interferometer; PID and Piezo Servo, Proportion Integration Differentiation controller and the driver to the PZT2 for locking the interferometer.

**Supplementary Note 6. Theoretical derivation of the shot noise sensitivity**

To explore the detection limit of the MIM system, we theoretically calculate the displacement sensitivity of our system at the shot noise limit. The response function between the PDH error signal and the membrane motion can be understood as follows: As a result of the coupling among the input field, the optical cavity and the membrane, the displacement of membrane is transformed into the phase variation of the reflected probe light. The beat between the sidebands of phase-modulated signal and the carrier in the reflection field is captured by a photodetector[10]. After modulation and demodulation through the PDH circuit, the displacement signal is revealed as the PDH error signal. Since the feedback bandwidth of our lock loop is lower than the spectral frequency we measured, the derivation will be treated for an open-loop PDH error signal.

Firstly, we construct the relationship between the membrane displacement and the reflected light field. Considering the phase modulation of the probe field with the EOM, the input field can be described as[11]

$$E_{\mathrm{in}}(t) = E_0 e^{i\alpha\, sin(\Omega_0 t)} = E_0 \sum_n J_n\,(\alpha) e^{in\Omega_0 t}. \tag{1}$$

where $\alpha$ is the phase modulation depth and $\Omega_0$ is the phase modulation frequency of the EOM. $J_n$ is the $n^{\mathrm{th}}$-order Bessel function of the first kind.

For a symmetrical, lossless, high-finesse cavity, the total amplitude decay rate $\kappa = 2\kappa_{\mathrm{m}}$ where $\kappa_{\mathrm{m}}$ is the amplitude decay rate through the input/output mirror. When the input field is resonant with the cavity (Δ=0, the detuning between the input field and the cavity), the first-order motion equation of the field in the cavity letting $a(t) = \langle a \rangle + \delta a(t)$ is modelled as[12]

$$\dot{a}(t) = -\kappa a(t) + i\delta\omega_c(t)\langle a \rangle + \sqrt{\kappa} E_{\mathrm{in}}(t), \tag{2}$$

in which $\langle a \rangle = \frac{E_0 J_0(\alpha)}{\sqrt{\kappa}}$ and $\delta\omega_{\mathrm{c}}(t)$ is the phase shift of the reflection field. The transmitted and reflected fields can be expressed as $E_{\mathrm{out}}(t) = \sqrt{\kappa}a(t)$ and $E_{\mathrm{ref}}(t) = -E_{\mathrm{in}}(t) + \sqrt{\kappa}a(t)$.

After the Fourier transform to both sides and under the condition of $\Omega_0 \gg \kappa$, we get

$$a(\Omega) = \frac{\sqrt{\kappa}}{\kappa + i\Omega}\left(2\pi\sum_n J_n(\alpha)\delta(\Omega - n\Omega_0) + \frac{i\delta\omega_c(\Omega)}{\kappa}J_0(\alpha)\right)E_0, \qquad (3.1)$$

$$E_{\mathrm{ref}}(\Omega) = \left[2\pi\left(\frac{\kappa}{\kappa + i\Omega} - 1\right)E_0\sum_n J_n(\alpha)\delta(\Omega - n\Omega_0) + \frac{i}{\kappa + i\Omega}\delta\omega_c(\Omega)J_0(\alpha)\right]E_0. \qquad (3.2)$$

Here, the Fourier transform is defined as: $f(\Omega) = \int_{-\infty}^{+\infty} f(t)\cdot e^{-i\Omega t}dt$ and the inverse Fourier transform is defined as $f(t) = \frac{1}{2\pi}\int_{-\infty}^{+\infty} f(\Omega)\cdot e^{i\Omega t}d\Omega$.

To consider the periodic motion of the membrane,$\delta\omega_c(t) = \delta\omega_0\, cos(\Omega_{\mathrm{m}}t) = g_m\eta_{xy}\delta z_{m,0}\, cos(\Omega_{\mathrm{m}}t)$ in which $g_m$ is the optomechanical coupling coefficient and $\eta_{xy}$ the overlap coefficient depending on the spatial overlap between of the mechanical and the optical spatial modes. Through the inverse Fourier transform, we get

$$E_{\mathrm{ref}}(t) = \sum_n \left(\frac{\kappa}{\kappa + in\Omega_0} - 1\right)E_0 J_n(\alpha)e^{in\Omega_0 t} + i\delta\omega_0 J_0(\alpha)\frac{1}{2}\left(\frac{e^{i\Omega_{\mathrm{m}}t}}{\kappa + i\Omega_{\mathrm{m}}} + c.c.\right)E_0. \qquad (4)$$

Next, we describe the process by which the reflected light enters the detector and outputs as an electrical signal through the PDH circuit. The output electrical, called the error signal, is used for locking the wavelength of the laser and for analyzing the motion of the membrane.

The error signal is obtained by mixing the RF detector output and the local oscillator (LO) through the mixer. The RF part of the reflected field is defined as

$$\delta|E_{\mathrm{ref}}(t)|^2 \equiv |E_{\mathrm{ref}}(t)|^2 - \langle|E_{\mathrm{ref}}(t)|^2\rangle, \qquad (5)$$

where $\langle|E_{\text{ref}}(t)|^2\rangle \approx |E_0|^2\left(1-\left(\frac{4\kappa_1\kappa_2}{\kappa^2}\right)J_0^2(\alpha)\right)=\frac{\langle i\rangle}{A}$ and A (unit: A/W) is the photodetector's responsivity.

Assuming there is no conversion loss for the mixer between the photocurrent and the LO signal ($\Omega_0 \sim 3MHz$), the resulting photocurrent would be

$$\epsilon(t)=\frac{4J_0(\alpha)J_1(\alpha)g_{\text{m}}\eta_{xy}}{\kappa[1-J_0^2(\alpha)]}\langle i\rangle g_{\text{m}}\eta_{xy}\delta z_{\text{m},0}\sqrt{\frac{1}{1+\left(\frac{\Omega_{\text{m}}}{\kappa}\right)^2}}\cos\left(\Omega_{\text{m}}t-\tan^{-1}\left(\frac{\Omega_{\text{m}}}{\kappa}\right)\right). \tag{6}$$

The relationship between the error signal and the membrane motion signal is then expressed as

$$G_{\epsilon,z_{\text{m}}}(\Omega)\equiv\frac{\epsilon(\Omega)}{\delta z_{\text{m}}(\Omega)}=\frac{4J_0(\alpha)J_1(\alpha)g_{\text{m}}\eta_{xy}}{\kappa[1-J_0^2(\alpha)]}\langle i\rangle\sqrt{\frac{1}{1+(\frac{\Omega}{\kappa})^2}}e^{-i\tan^{-1}(\frac{\Omega}{\kappa})}. \tag{7}$$

Here, we assume that the probe field matches the cavity mode perfectly. However, in reality the mode matching is affected by the spatial mode and the polarization of the input beam. We divide the incident light into $\vec{E}_{in}^{a}$, which matches the cavity, and $\vec{E}_{in}^{b}$, which reflects directly.

The coupling efficiency $\boldsymbol{\xi}$ and the photocurrent $\langle i\rangle$ can be expressed as[12]

$$\xi^2\equiv\frac{\langle|E_{\text{in}}^a(t)|^2\rangle}{\left\langle\left|E_{\text{in}}^a(t)\right|^2\right\rangle+\left\langle\left|E_{\text{in}}^b(t)\right|^2\right\rangle}=\frac{\langle P_{\text{in}}^a\rangle}{\langle P_{\text{in}}\rangle}, \tag{8.1}$$

$$\langle i\rangle=\langle i_a\rangle+\langle i_b\rangle=A\left|E_{\text{in},0}^a\right|^2\left[1-J_0^2(\alpha)+\frac{1-\xi^2}{\xi^2}\right]. \tag{8.2}$$

Referring to Eq. (7), the PDH response function is given as

$$G_{\epsilon,z_{\text{m}}}(\Omega)=\frac{4J_0(\alpha)J_1(\alpha)g_{\text{m}}\eta_{xy}}{\kappa[1-J_0^2(\alpha)+\frac{1-\xi^2}{\xi^2}]}\langle i\rangle\sqrt{\frac{1}{1+(\frac{\Omega}{\kappa})^2}}e^{-i\tan^{-1}(\frac{\Omega}{\kappa})}. \tag{9}$$

Using the photocurrent noise expression, $S_{\epsilon}^{\text{shot}}(\Omega) = 2e\langle i\rangle = 2eA\langle P_{\text{ref}}\rangle$, where $P_{\text{ref}}$ is the reflected light power, we obtain the shot-noise-equivalent displacement as

$$\sqrt{S_{z_{\text{m}}}^{\text{shot,PDH}}(\Omega)} = \frac{\sqrt{S_{\epsilon}^{\text{shot}}(\Omega)}}{G_{\epsilon,z_{\text{m}}}(\Omega)} = \frac{\kappa[1 - J_0^2(\alpha) + \frac{1-\xi^2}{\xi^2}]}{4J_0(\alpha)J_1(\alpha)g_{\text{m}}\eta_{xy}}\sqrt{\frac{2e}{A\langle \text{P}_{\text{ref}}\rangle}}\sqrt{1 + (\frac{\Omega}{\kappa})^2}. \quad (10)$$

Now we substitute the parameters of the MIM system into Eq. (10). The parameters are given as

$$\{\kappa, \xi^2, \alpha, A, g_{\text{m}}, \eta_{xy}, \langle P_{\text{ref}}\rangle\} = \{2\pi \times 210\ \text{kHz}, 0.36, 0.1, 0.65\text{A/W}, 0.045\text{Hz/m}, 1, 2.6\mu\text{W}\} \quad (11)$$

It should be noted that $g_m$ is the maximum value ($g_{\text{m}}^{\max} = 2\mathcal{R}_{\text{m},\lambda}g_0 = \frac{4\pi\mathcal{R}_{\text{m},\lambda}c}{L\lambda}$) where $\mathcal{R}_{\text{m},\lambda}$ (~0.38) means the reflectance of the thin film for the light at the wavelength $\lambda$ (=1064 nm). The overlap coefficient $\eta_{xy}$ ~1 for the ideal case where the incident beam spot is located in the center of the film in the (1,1) mode.

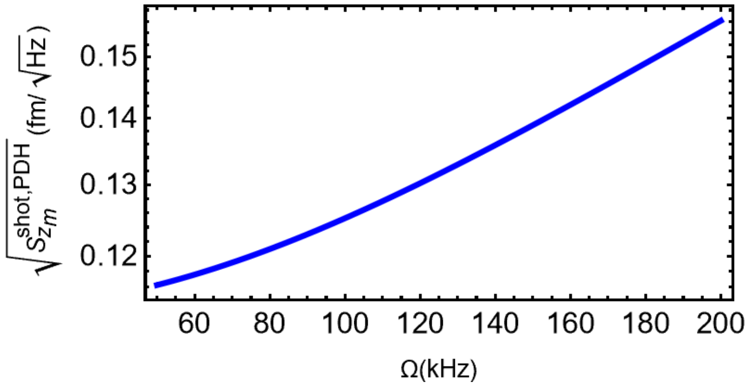


**Fig. 7 | Shot-noise-equivalent displacement as a function of the phase-modulated frequency.**

In our experiment, the frequency (~68 kHz) with the highest resonance responsivity of the membrane under the atmospheric pressure was used. As shown in Fig. 7, for the resonance frequency at 68 kHz, the shot-noise-limited displacement sensitivity is 0.12 fm/$\sqrt{Hz}$.

## Supplementary Note 7. Limits on broadband measurement

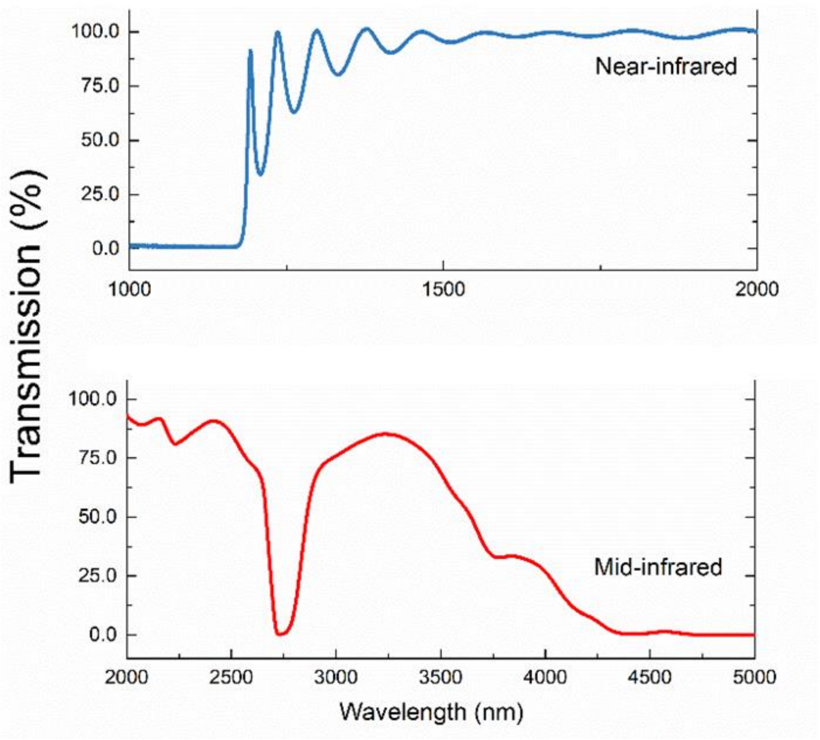


**Fig. 8 | Transmission spectra of the resonant cavity.** The spectra are measured with a commercial infrared spectrometer (spotlight400, PerkinElmer; resolution of 1 $cm^{-1}$).

We should mention that the simultaneous spectral bandwidth (2 nm or 240 GHz) is currently limited by the laser source we used, i.e., the EO combs, instead of the concept we proposed or the mirror-in-the-middle (MIM) system.

The transmission spectra of the MIM cavity are shown in Fig. 8. The spectrum spans from 1200 nm to 4500 nm. The cavity is designed to be resonant with the probe laser at 1064 nm, which explains the cutoff at 1200 nm. Currently, the cavity mirrors are not for mid-infrared wavelengths above 4500 nm. A solution to this issue is to separate the dual combs and probe beams spatially. To be more specific, the dual-comb excitation beam spatially coincides with the probe on the MIM membrane but does not pass through the resonant mirror, as depictured in Fig. 9. In our experiment, we notice that making the excitation and probe beams propagate collinearly (Fig. 9a) is

unnecessary. As long as the molecules around the MIM membrane were excited, we could observe the interferometric signal sensed by the probe beam. Therefore, shining the dual combs at oblique incidence (Fig. 9b) will avoid the limitations of the cavity mirrors on broadband mid-infrared measurement.

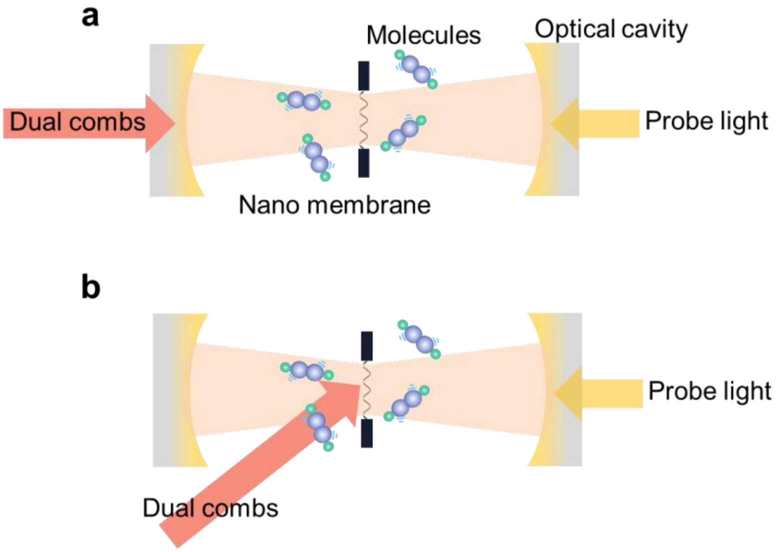


**Fig. 9 | Configurations of DCOS. a** collinear configuration; **b** DCOS with crossed beams.

## Supplementary References


1. Shang, Z. et al. Quartz-enhanced photoacoustic NH(3) sensor exploiting a large-prong-spacing quartz tuning fork and an optical fiber amplifier for biomedical applications. *Photoacoustic* **26**, 100363 (2022).
2. Fu, L. et al. Small-volume highly-sensitive all-optical gas sensor using non-resonant photoacoustic spectroscopy with dual silicon cantilever optical microphones. *Photoacoustics* **27**, 100382 (2022).
3. Xiao, H. et al. Ultra-sensitive ppb-level methane detection based on NIR all-optical photoacoustic spectroscopy by using differential fiber-optic microphones with gold-chromium composite nanomembrane. *Photoacoustics* **26**, 100353 (2022).
4. Ma, Y. et al. Highly sensitive acetylene detection based on multi-pass retro-reflection-cavity-enhanced photoacoustic spectroscopy and a fiber amplified diode laser. *Opt. Express* **27**, 14163 (2019).
5. Hayden, J. Giglio, M. Sampaolo, A. Spagnolo, V. & Lendl, B. Mid-infrared intracavity quartz-enhanced photoacoustic spectroscopy with pptv – Level sensitivity using a T-shaped custom tuning fork. *Photoacoustics* **25**, 100330 (2022).
6. Wang, Z. et al. Ultrasensitive photoacoustic detection in a high-finesse cavity with Pound-Drever-Hall locking. *Opt Lett.* **44**(8), 1924 (2019).
7. Wang. Z et al. Doubly resonant sub-ppt photoacoustic gas detection with eight decades dynamic range. *Photoacoustics* **27**, 100387 (2022).
8. Tomberg, T. Hieta, T. Vainio, M. & Halonen, L. Cavity-enhanced cantilever-enhanced photo-acoustic spectroscopy. *Analyst* **144 (7)**, 2291-2296 (2019).
9. Black, E. D. An introduction to Pound-Drever-Hall laser frequency stabilization. *Am. J. Phys.* **69**, 79 (2001).
10. Drever, R. W. P. et al. Laser phase and frequency stabilization using an optical resonator, Appl. Phys. B 31, 97-105 (1983).

11. Bjorklund, G. C. Levenson, M. D. Lenth, W. & Ortiz, C. Frequency Modulation Spectroscopy, *Appl. Phys. B* **32**, 145-152 (1983).

12. Wilson, D. J. “Cavity optomechanics with high-stress silicon nitride films”, thesis, California Institute of Technology, Pasadena, CA(2012).

13. Wei, X. Sheng, J. Wu, Y. Liu, W. & Wu, H. Twin-beam-enhanced displacement measurement of a membrane in a cavity. *Appl. Phys. Lett.* **115**, 251105 (2019).
